# Supplementary figures and images for: Pursuit of chlorovirus genetic transformation and CRISPR/Cas9-mediated gene editing
Source: PLoS One. 2021 Oct 21;16(10):e0252696. doi: 10.1371/journal.pone.0252696 (PMC8530361; doi:10.1371/journal.pone.0252696)

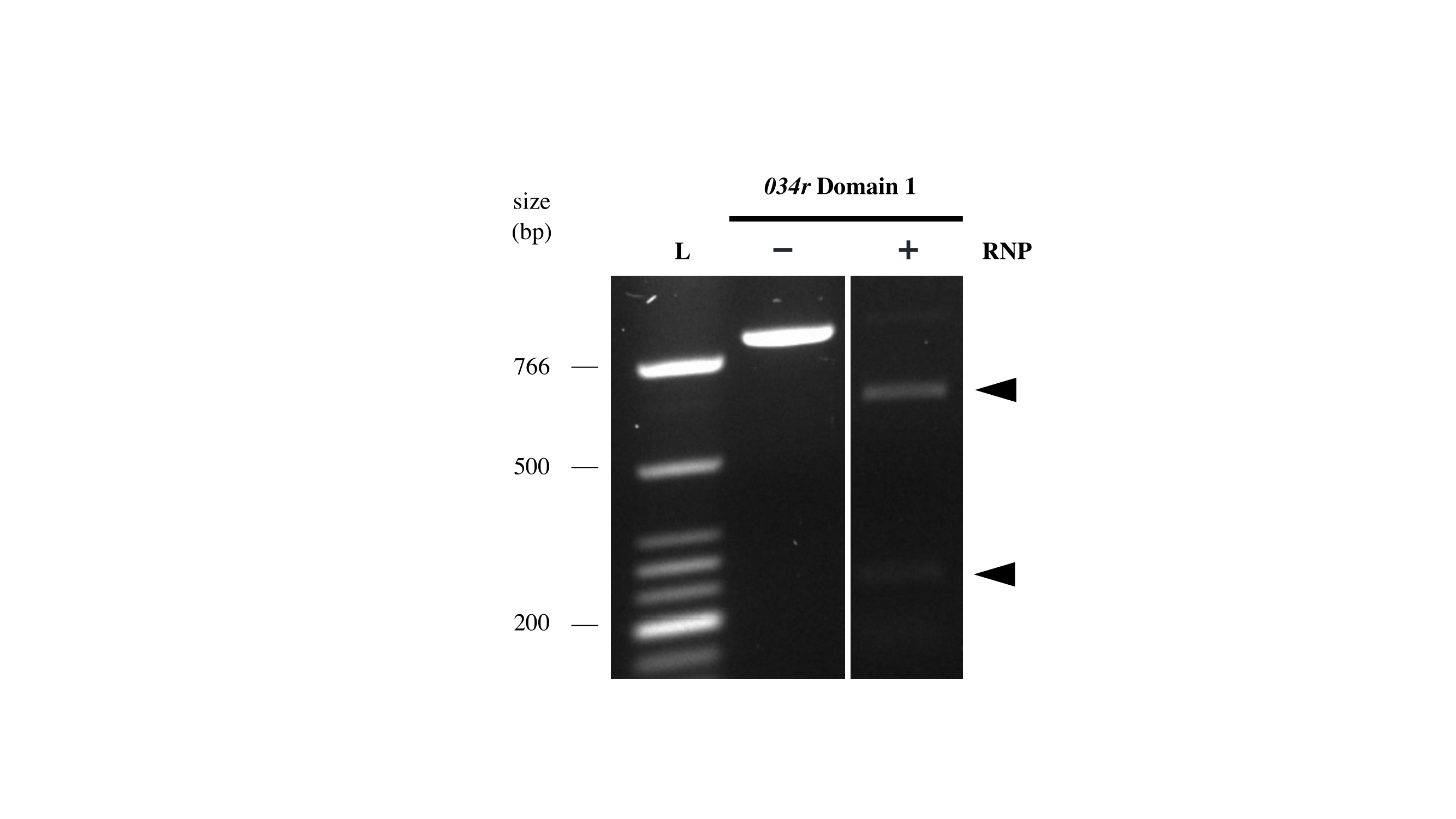

Supplement: S1 Fig — The target 034r locus from NC64A virus CA-4B was PCR-amplified and incubated with preassembled Cas9 and sgRNA RNP complexes in vitro. The complete in vitro cleavage of the target locus confirmed active RNP formation. PCR product (835 nucleotides) was amplified using primers upstream and downstream of domain 1. Arrowheads indicate cleaved products. L, molecular size ladder. (TIFF) [file pone.0252696.s001.tiff]

**Fig 5A (Left)**

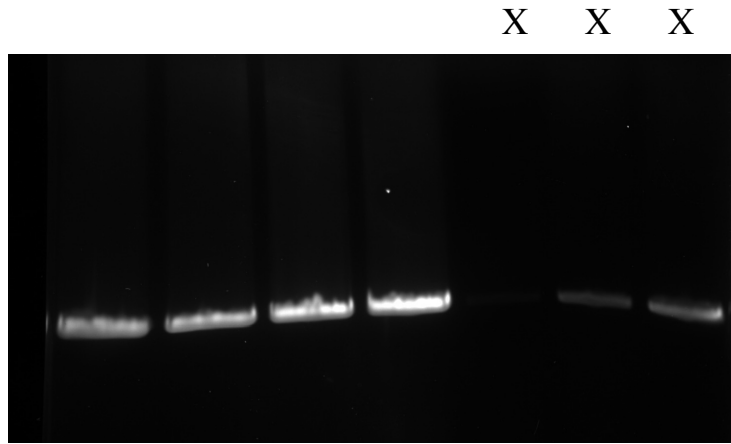

**Fig 5A (Right)**

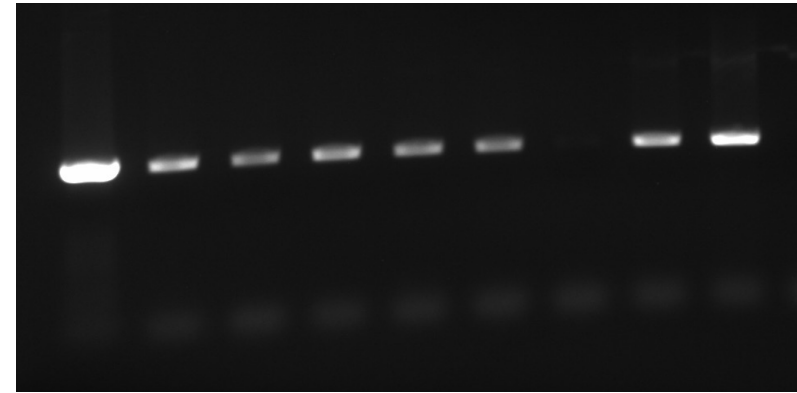

**Fig 5B**

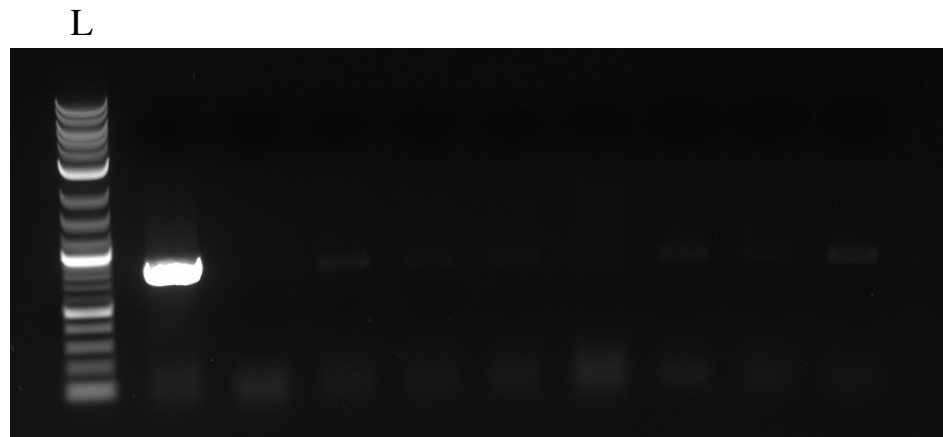

**Fig S1**

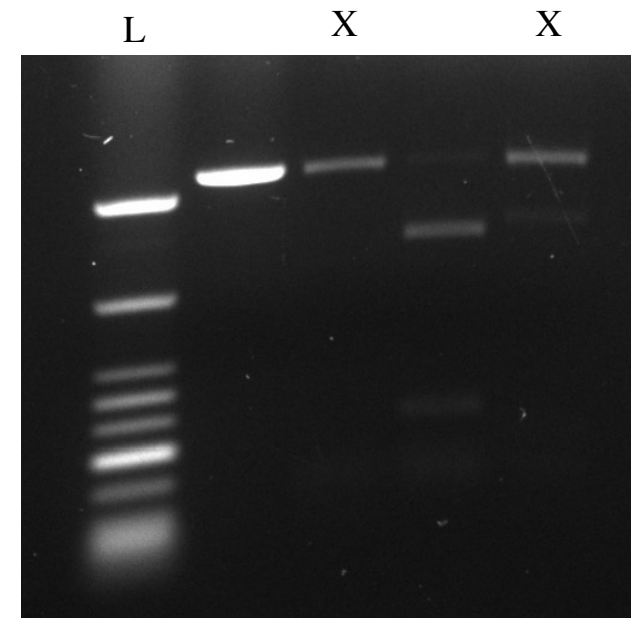

Supplement: S1 Raw images — (PDF) [file pone.0252696.s002.pdf]
