# Supplementary material for: Pursuit of chlorovirus genetic transformation and CRISPR/Cas9-mediated gene editing
Source: PLoS One. 2021 Oct 21;16(10):e0252696. doi: 10.1371/journal.pone.0252696 (PMC8530361; doi:10.1371/journal.pone.0252696)
Supplement: S1 Dataset — (PDF) [file pone.0252696.s003.pdf]

Fig 1B. Data set.

	Number of NC64A cells (1x10^8/mL)					Number of NC64A cells (1x10^8/mL)				
Cefotaxime (mg/L)	Day 4 - Sample 1	Day 4 - Sample 2	Day 4 - Sample 3	Day 4 - AVG	Day 4 - SD	Day 8 - Sample 1	Day 8 - Sample 2	Day 8 - Sample 3	Day 8 - AVG	Day 8 - SD
0	5.53	5.4	6	5.643333333	0.315647483	6.31	6.4	6.7	6.47	0.204205779
100	5.7	5.5	4.9	5.366666667	0.4163332	6.74	6.7	6.98	6.806666667	0.151437556
200	5.39	4.95	5.17	5.17	0.22	6.8	7.56	6.87	7.076666667	0.420039681
300	5.96	6.1	5.9	5.986666667	0.102632029	6.34	6.1	6.35	6.263333333	0.141539158
400	5.55	5.6	6.39	5.846666667	0.471204131	5.95	5.75	6.2	5.966666667	0.225462488
500	5.85	5.21	5.1	5.386666667	0.405010288	6.58	6.4	6.13	6.37	0.226495033
600	5.29	6.1	5.15	5.513333333	0.512867754	5.58	5.3	4.9	5.26	0.34176015
700	5.3	5.34	5.7	5.446666667	0.220302822	5.89	5.13	6.1	5.706666667	0.510326693
800	5.11	5.22	6.1	5.476666667	0.542617115	6.36	6.24	6.69	6.43	0.233023604
900	4.53	4.1	4.45	4.36	0.228691933	5.53	5.3	5.1	5.31	0.215174348
1000	4.66	4.1	4.6	4.453333333	0.307462735	5.9	5.4	6.23	5.843333333	0.417891533

Fig 1C. Data set.

Hygromycin (mg/L)	Number of NC64A cells (1x10^8/mL)			AVG	SD
6	6.79	6.3	4.81		1.03122904
8	4.4	4.1	5.12		0.524213697
10	4	3.54	4.2		0.338427737
12	2	2.3	1.87		0.220529665
14	1.5	2.32	2.21		0.445084262
16	1	1.2	1.45		0.225462488
18	0.35	0.57	0.95		0.303534732
20	0	0	0		0
23	0	0	0		0
26	0	0	0		0
