# Supplementary material for: Pursuit of chlorovirus genetic transformation and CRISPR/Cas9-mediated gene editing
Source: PLoS One. 2021 Oct 21;16(10):e0252696. doi: 10.1371/journal.pone.0252696 (PMC8530361; doi:10.1371/journal.pone.0252696)
Supplement: Attachment — Submitted filename: Response to Reviewers.docx [file pone.0252696.s004.docx]

**Two major concerns raised by the reviewers:**

1. Frequency of obtained gene edited events: The reviewer 1 is convinced that the obtained mutations are indeed achieved by Cas9-mediated gene editing but more information such as efficiency and target sequences should be provided. The reviewer 2 raises concerns whether the gene edited events could be obtained by natural mutations. Thus, please provide information to exclude the occurrence of natural mutation or explain why this is indeed caused by gene editing.

Response: From the thousands of publications reporting the repair of double-stranded DNA breaks caused by CRISPR/Cas9 in eukaryotic cells, it is clear that the cellular repair systems initiate repair at the cut site and that the vast majority of repairs involve small nucleotide deletions or additions – most within two or three nucleotides of the cleavage site. The two mutations discovered in our studies (one nucleotide deletion and one nucleotide insertion) are precisely at the Cas9/sgRNA targeted cleavage site. With present data, it would, of course, be difficult to precisely calculate the odds of obtaining two “natural” mutations at the cut site of the *034r* gene. We can say however that there were no other mutations found in the total of approximately 30,000 nucleotides we sequenced in the PCR amplification products from all 56 viruses we selected in the relevant experiments we report in the present manuscript. Given that we found two mutations at the cleavage site, and none elsewhere, the minimum probability of finding both mutations at the same site within ~500 nucleotides would, minimally, be the product of that probability (i.e., ~1/500 X ~1/500 or ~1/250000). Thus, even from this elementary analysis of the data, it is clear that it is exceedingly unlikely that we would have observed the two mutations we did observe as the result of “natural” (i.e., spontaneous) mutations. Because a rigorous assessment of all the factors involved in establishing an accurate mutation rate in these particular experiments would be highly challenging and because the experiments were not designed to make such determinations, we believe it is fair and intellectually honest for us to say in simple terms that the likelihood of the mutations being the result of Cas9/sgRNA cleavage site repair are very high (lines 475-484 in the revised Manuscript)*.*

2. Writing style and format of the article: Although the results of this manuscript did not achieve the original goal by establishing the reproducible gene editing method, there are still many positive aspects reported in this manuscript. I agree with the reviewer 1 that the abstract should describe more results obtained during the method development than focusing on the negative results. As this is a manuscript for a method development, some parts of methods can be described as part of results.

Response: We agree with the reviewer’s and Editor’s suggestions and have rearranged and revised the abstract to emphasize more of the positive results achieved in our studies. Likewise, we have rearranged the order of presentation of experimental data and moved significant portions of the Methods section into the Results section of the manuscript.

**Comments from the Editor:**

1. Please ensure that your manuscript meets PLOS ONE's style requirements, including those for file naming.

Response: The appropriate edits have been made so the manuscript is aligned with PLOS ONE’s style requirements.

2. PLOS ONE now requires that authors provide the original uncropped and unadjusted images underlying all blot or gel results reported in a submission’s figures or Supporting Information files. This policy and the journal’s other requirements for blot/gel reporting and figure preparation are described in detail at https://journals.plos.org/plosone/s/figures#loc-blot-and-gel-reporting-requirements and https://journals.plos.org/plosone/s/figures#loc-preparing-figures-from-image-files. When you submit your revised manuscript, please ensure that your figures adhere fully to these guidelines and provide the original underlying images for all blot or gel data reported in your submission. See the following link for instructions on providing the original image data: https://journals.plos.org/plosone/s/figures#loc-original-images-for-blots-and-gels.

Response: File “S1_raw_images” was previously submitted as a Supporting Information file with the original manuscript as instructed by PLOS ONE guidelines. This file is a single PDF that contains all the original blot and gel images contained in the manuscript’s main figures and supplemental figure. Each gel image is labeled to clearly annotate the loading order, identify the experimental samples, and to specify which figure panel was generated from that original image.

In your cover letter, please note whether your blot/gel image data are in Supporting Information or posted at a public data repository, provide the repository URL if relevant, and provide specific details as to which raw blot/gel images, if any, are not available. Email us at plosone@plos.org if you have any questions.

Response: This is addressed in the cover letter.

3. We note that you have included the phrase “data not shown” in your manuscript. Unfortunately, this does not meet our data sharing requirements. PLOS does not permit references to inaccessible data. We require that authors provide all relevant data within the paper, Supporting Information files, or in an acceptable, public repository. Please add a citation to support this phrase or upload the data that corresponds with these findings to a stable repository (such as Figshare or Dryad) and provide and URLs, DOIs, or accession numbers that may be used to access these data. Or, if the data are not a core part of the research being presented in your study, we ask that you remove the phrase that refers to these data.

Response: “data not shown” has been removed (lines 389 and 509 in the revised Manuscript).

4. Please amend your manuscript to include your abstract after the title page.

Response: Abstract was added to the manuscript after the title page.

5. Please include captions for your Supporting Information files at the end of your manuscript, and update any in-text citations to match accordingly. Please see our Supporting Information guidelines for more information: [http://journals.plos.org/plosone/s/supporting-information](about:blank).

Response: Supporting Information file “Fig S1” has been moved to the end of the manuscript (lines 609-616 in the revised Manuscript).

**Reviewer #1**

Engineering DNA viruses of green algae could be important biotechnology to solve the environmental problem such as algae bloom. Here ,the attempt is to transform CRISPR/Cas9 into the algal host Chlorella variabilis NC64A and infected with Chloroviruses. Theoretically, genome edited Chlorovirus can be obtained. The pre-requested technique is the ability to transform Chlorella variabilis NC64A. The authors tried several transformation methods including electroporation of RNP, transformation after cell-wall digestion, silicon carbide whiskers, cell-penetrating peptides transformation of RNP and Agrobacterium transformation of DNA. Most of these transformation methods were unsuccessful to obtain mutated virus, however, two mutated viruses were identified from macerozyme-digested algae. This result demonstrated CRISPR/Cas9-mediated gene editing of chlorovirus genome is possible.

This article has clear introduction of background knowledge. The authors also properly discussed the results they obtained. Overall, the experimental design is logical to achieve their goal. Most importantly, they obtained two mutated viruses with different indels, indicating this experiment is success in certain level.

Notably, I think the authors did not fully aware of the differences between a research article and an article of methodology. Two issues are likely related to this problem: 1-“Methods” part is a mixture with “results and methods”; 2-unable to duplicate these results of edited chloroviruses.

Current “method” part is very difficult to read, I am sorry to say. For a research article published on PLOS ONE, the procedure of experimental designs and first figure are put at “Method” part before “Results” section could be common. But for a methodology research, development is part of results. Please consider to put only “methods” that mostly followed previous references with minor modifications in the “methods”.

Response: The reviewer makes a valid point. In response we have transferred a significant portion of the Methods section into the Results section of the manuscript in a manner that we trust makes the understanding of our experimental approaches and procedures easier for readers to follow.

Second, the authors used a negative way to describe their result: “they were unable to duplicate these results and therefore unable to achieve a reliable system to genetically edit chloroviruses” in the abstract and most of times, besides in the discussion. However, performing gene editing in non-model organisms is not an easy task, it is even more challenge to edit viral genome of this non-model organism. I still think this research has its breakthrough. For this, I would suggest the authors try to describe this result from a positive aspect. Also, the results from these two mutated viruses could be more solid. Here are some suggestions: - These mutants may come from rare events, but how rare it is? How many plagues were sequencing eventually? How many were from macerozyme digestion? How many were from rest of other enzymes?

Response: In response to the reviewer’s (and Editor’s) comments we have now inserted additional information and discussion (lines 475-484 in the revised Manuscript) that provides the reader with a better sense of the rarity of the mutations and the extremely unlikely probability that these indels are the result of spontaneous mutations. We have also added the information that after finding the two mutations in our first experiment we repeated the experiment several times and, ultimately, examined DNA from over 50 independent viral plagues obtained using the same protocol. Because of the initial success was with using macerozyme-treated cells, all of our experimental replications were performed using only such cells. We have inserted a note in the text (lines 475-476 in revised Manuscript) that the same experimental protocol was used for all repeats of our experiment using the one particular Cas9/RNP complex that provided initial success.

- Fig 3C, please also show chromatograph picture of Sanger sequencing and mark these indel and PAM information.

Response: The DNA sequencing was performed by a commercial organization and the chromatograph is not available to us. However, as we note in the text, the sequencing was performed in triplicate with the same results in each instance.

- Besides therapeutic purpose such as preventing virus infection, how many success cases of manipulating viral genome by CRISPR?

Response: Yes, there have been many advances with CRISPR/Cas9 system-based gene editing in virology including its applications in antiviral vaccine development, especially in large DNA viruses of animals. The CRISPR/Cas9 system has been successfully applied to the gene editing of many different virus species (*Teng et al*., 2021), mainly focusing on antiviral therapy, functional study of viral virulence factors, and the reconstitution of commonly used viral vectors for genetically engineered vaccine development. These advances are now discussed in our manuscript to provide additional perspectives to readers (lines 527-532 in the revised Manuscript).

Other suggestions:

Fig 1 is experimental design should not only appear only at introduction.

Response: Thank you for the suggestion; we have moved Figure 1 (now Fig 6) to the newly expanded Results section.

Table 1. Would be better if you can show the quantification results of cell growth inhibition by enzyme.

Response: Unfortunately, we did not make measurements of the diameters of the zones of cell growth inhibition in these experiments aimed at providing rough estimates of cell growth inhibition by various cell wall-disrupting enzymes. The relatively small size of the zones of inhibition makes truly accurate measurements difficult. We have, however, now inserted a more complete description in Table 1 of what is meant by each symbol we employed (line 411 in the revised Manuscript). That is: +++, complete cell growth inhibition; ++, moderate growth inhibition; +, slight growth inhibition; -, no growth inhibition.

Fig 1. The cleavage site (green triangle) should be between TTTT and ATCTGG, three nucleotides away from PAM, also Fig 2C. This actually increases the reliability of the result that mutations were indeed came from Cas9 cleavage, the 1-nt deletion and 1-nt insertion are all adjacent to the cleavage site, two very common insertion and deletion.

Response: Thank you for pointing out this error in our Figure 1 (now Fig 6). It has been corrected and references to this precise cut site have now been added to the revised Manuscript (lines 478-482 and 538-540) – and also emphasize the fact that the indels are located at this exact site.

“sgRNA” to “sgRNA or crRNA”. Cas9 uses an artificial sgRNA, Cpf1 uses its native crRNA. They cannot use the same sgRNA. The target site of Cpf1 is TTTV, followed by protospacer target sites at downstream of TTTV PAM. What is the target sequence of Cpf1?

Response: Although we tested the ability of Cpf1 to create mutations in chlorovirus CA-4B, we obtained no positive results and, therefore present no data in the manuscript. Therefore, we have eliminated all reference to its use from the revised manuscript.

Line 490 (now line 536 in the revised Manuscript) “we were unable to produce a reliable transformation system”. The description here is not complete wrong but can be more positive. I tried to image the difficulty of obtaining the mutated virus. Is that correct that mutated viral genome may not be enveloped into mutated envelop? CRISPR/Cas9 is not an efficient nuclease at short-time window in many organisms. If only a small proportion of viral genome is mutated during their duplication in host cell, only an even smaller proportion of mutated viral genome is enveloped into mutated viral capsid, is that possible?

Response: We do not think this would be an issue for the artificially mutated genomes (i.e., like those created in this study) to not be packaged in the virus particle because all of the spontaneously generated, mutated genomes selected in earlier studies are packaged into viruses that both efficiently infect and reproduce in host cells.

Line 383 (now line 467 in the revised Manuscript) “Two independent plaques”

Is that possible these two virus mutants came from the same host cell?

Response: This is a very difficult question to answer – especially given the observed low success rate in obtaining mutant viruses. In theory, considering the average burst size per cell is ~1000 virus particles, this is a possibility. Clearly, the two DNA sequences were not the result of one mutation that was replicated into two identical DNA virus strands given that they bear two distinctly different mutations (i.e., they represent two truly independent mutation events occurring during repair of two separate DNA strands). Indeed, if uptake of Cas9 RNPS is a rare event, then the likelihood of a single cell producing two mutational events increases substantially. However, the best we can say is the following statement now inserted into the manuscript (lines 475-484 in the revised Manuscript): (Whether the two mutations observed occurred in a single NC64A cell following the rare uptake of Cas9 RNPs or in two independent cells is not possible to determine).

Line 391 “Many attempts to obtain additional examples of gene editing with this particular Cas9/sgRNA RNP failed.”

Please specify. How many batches of experiment were performed? How many plagues were analyzed? How many plagues are wildtype 034r gene?

Response: Ten additional attempts to obtain examples of gene editing with this particular Cas9/sgRNA RNP failed. These experiments included the sequencing of more than 50 plaques (line 479 in the revised Manuscript).

In Fig 5C, resolution of plate picture is too low to show colonies.

Response: Unfortunately, we cannot improve the resolution.

Fig 6.” imaged through green, red, and GFP channels (from left to right).” The description of respective images is not clear. Frankly speaking, I am highly suspicious to the fluorescent signals of GFP. These signals overlapped nearly completely with Red channel (is this autofluorescence signal, from chloroplast?). Unless the GFP-GUS proteins have chloroplast translocation signal peptide, otherwise it is unlikely overlapped with chloroplast. In plant cells, GFP can be observed in cytosol and nucleus, but not in chloroplast and vacuole. Also, GUS usually is a much better reporter than GFP. Besides, please confirm white light image. If all cells here are all with GFP signals, the transformation rate is unreasonably high. The presence of the gfp-gusA gene PCR could come from agrobacterium.

Response: Figure 6 (now Fig 3) legend had been updated to address the reviewers concerns and to provide clarity of the confocal microscopy images (lines 392-398 in the revised Manuscript). The reviewer also fairly questions the origins of the gfp-gusA gene PCR. As stated in the text, to rule this out, we subcultured these colonies on nutrient-rich LB media to support any residual Agrobacterium growth – and, as also stated in the manuscript, no bacteria were detected after three weeks of incubation under optimal growing conditions.

**Reviewer #2**

1. The two mutants obtained was likely to result from Cas9 cleavage and following NHEJ. However, the possibility of natural mutation cannot be excluded based on the provided data. The authors used two types of antibodies for wildtype and mutant surface structure, which may select the viruses with natural mutation in the glycosyltransferase. To exclude the possibility, the authors should demonstrate the mutation was introduced only in the targeted site at significantly higher ratio than natural mutation. Full length of 034r gene should be sequenced from all antibody-selected viruses and mutation rate in the gene should be calculated.

Response: We have addressed this matter above in earlier comments to both the editor and Reviewer 1 and believe the revisions we have made in response provide the firmest and fairest conclusions that can be drawn at this time. Please refer to the pertinent parts of those responses.

2. Agrobacterium-mediated transformation system is promising, but not closely related to the reverse-genetics system in chlorovirus. If the authors had used the system for gene-editing, it could be included. Otherwise, this part should be removed from this article.

Response: We have chosen to present the *Agrobacterium*-mediated cell transformation experiments because, indeed, the results are promising and may serve as a significant starting point for other researchers who wish to genetically modify NC64A or other related algae. Knowing that the practical challenge may lie in preventing gene silencing and knowing that there are other laboratories who have successfully dealt with this (significant) challenge in various ways, may provide others an appropriate measure of encouragement to proceed (with due caution learned from the present studies).

Below are minor comments.

1. In method section, order of information should be consistent with the order of presented results. Used chemicals should be summarized in early part of the section.

Response: These are good suggestions and we have rearranged the methods accordingly. Please note, following the suggestion of the editor, we have condensed the Methods section significantly while providing expanded information regarding protocols in the Results section.

2. As the trial of homologous recombination failed, this part can be reduced. One sentence with “data not shown” is enough.

Response: Per PLOS ONE guidelines, authors are not allowed to use the phrase “data not shown”. Our modifications to this section have been discussed in an earlier Response above.

3. Table 1: Growth inhibition is ambiguous and subjective. The data should be presented in more quantitative and objective way.

Response: The authors agree this is an imprecise method for measuring the effects of various enzyme treatments on the disruption of the NC64A cell wall. However, as discussed above in response to comments by Reviewer 1, the present methods offer a rapid, practical, and dependable qualitative measure of the effectiveness of the enzymes in modifying cell wall integrity that, consequentially, affect the rate of cell growth. As we state in the text, the fact that heat denatured enzymes have no observable effect on the rate of cell growth compared to non-treated control cells provides a firm basis upon which to conclude that the observed results with the present assay provide a reasonable estimate of the effectiveness of the various enzyme treatments on cell growth – and, thus, indirectly, on the degree of cell wall disruption.

4. Figure 2 (now Fig 6 and Fig 7): This image includes experimental scheme and results. They should be separated.

Response: We agree. The one figure has been divided into two (Fig 6 and Fig 7 in revised Manuscript).

5. Line 472 (now 513 in the revised Manuscript): This paragraph is a kind of history. It is interesting but should be more organized for scientific paper.

Response: We agree that the paragraph provides what we consider useful history for those not completely familiar with the Chlorovirus field of research and its important (and sometimes practically useful) discoveries. A few edits have been made to hopefully improve its quality.

6. Experimental procedure is fairly overlapped in all sections. Small overlap should be allowed, as the article mainly described methods, but it is too much.

Response: As moted above, the manuscript has been significantly reorganized to place more of the experimental procedures in the Results section and make the manuscript more focused on methods. Hopefully, this has eliminated much of the overlap.
