# Supplementary material for: Pursuit of chlorovirus genetic transformation and CRISPR/Cas9-mediated gene editing
Source: PLoS One. 2021 Oct 21;16(10):e0252696. doi: 10.1371/journal.pone.0252696 (PMC8530361; doi:10.1371/journal.pone.0252696)
Supplement: Attachment — Submitted filename: Response to Reviewers.docx [file pone.0252696.s005.docx]

All referenced line numbers are from the document, “**Revised Manuscript with Track Changes**”.

**Comments from the Editor:**

1. As indicated by reviewer 1, please confirm the identity of gfp-gusA reporter vector and name consistency and accuracy (e.g. gfp-gusA or GFP-GUS reporter and using italics for Agrobacterium tumefaciens throughout the paper)

Response: We confirm the correction of the referenced *gfp-gusA* reporter and the usage of italics (Lines 161, 393, 412).

2. Please provide the evidence that GFP signal is derived from transformation event (Figure 3). Both reviewers concern the GFP signal could be autofluorescence considering that all cells shown have signals while no GUS activity could be detected. Please provide the image of negative controls and discuss the reason of no GUS signals. If not, please remove this figure.

Response: We have removed the original Figure 3 from the manuscript.

**Reviewer #1**:

The authors developed two transformation system: agrobacterium-mediated transformation and electroporation to transform algae Chlorella variabilis NC64A. The electroporation of in vitro synthesized CRISPR/Cas9 RNP complex together with Chloroviruses infection successfully generate two mutated Chloroviruses adjacent to the cleavage site of viral genome.

I think the current version description is reader friendly. The data support their main conclusions.

Line 390. I think it would be better if the authors can make a description why agrobacterium-transformation was not used for gene editing in this study due to the concern of gene silencing.

Response: The occurance of gene silencing in NC64A is not surprising given that it contains at least 375 homologs to genes involved in gene silencing (Rowe et al., 2013). Indeed, because of the apparent problems with gene silencing following *Agrobacterium*-mediated cell transformation, we did not consider it further for delivery of genes encoding Cas9 and sgRNA. Rather, we chose to employ *in vitro* assembled Cas9/sgRNA RNP complexes as described in the text. This explanation was added to the text (Lines 636-641).

*Rowe, J. M., Dunigan, D. D., Blanc, G., Gurnon, J. R., Xia, Y., & Van Etten, J. L. (2013). Evaluation of higher plant virus resistance genes in the green alga, Chlorella variabilis NC64A, during the early phase of infection with Paramecium bursaria chlorella virus-1. Virology, 442(2), 101-113*.

Line 36-37 In abstract “However, we were unable to duplicate these results and therefore unable to achieve a reliable system to genetically edit chloroviruses. Nonetheless…”. I guess the authors want to deliver a message that current method required further improvement. However, I think this point of view is fully discussed in the text already. “2 mutated viruses were obtained in total 56 plague” or “2 mutated viruses were obtained in only one of five replicates” are both correct description but give opposite impression to the readers

Response: To better satisfy the concern of the reviewer, we have reworded the sentence in question to read: “However, in ten subsequent experiments we were unable to duplicate these results and therefore unable to achieve a reliable system to genetically edit chloroviruses.” (Line 35).

Some paragraphs are not changed to the new orders accordingly:

Paragraph Line 156-161: “Cell wall-degrading enzymes” could be moved after Line 204.

Response: We agree. This paragraph has been moved to the described location (Lines 223-228).

Paragraph Line 582-591 “Finally, our results from Agrobacterium-mediated…” could be moved after Line 525.

Response: We agree. This paragraph has been moved to the described location (Lines 630-644).

Result HDR analysis, paragraph line 392-399

I think that although it is not necessary to show MscI digestion result in gel, but to evaluate a transformation or gene editing method, the analyzed sample numbers are critical. The MscI digestion to confirm HDR events might be fail to proof any success event, I think if the authors can show how many samples were confirmed in this part, we could have a better idea how far it is to reach successful HDR. Beside MscI digestion, the samples were sequenced should be included as well. If all MscI analyzed samples were sequenced, it is also fine.

Response: We believe the reviewer is referring to the paragraph under the sub header “No evidence for HDR events using ssODNs” (Lines 603-610). The number of MscI-digested-samples (38) is now provided (Line 663).

***Minor points:***

Line 370-371 gfp-gusA genes in pCAMBIA1303. As far as I checked, pCAMBIA1303 contain gus-gfp. Please confirm that pCAMBIA1303 and pCAMBIA1304 mentioned in this article are all correct.

Response: pCAMBIA1303 was a typo. All references of it have been replaced with pCAMBIA1304 (Lines 161, 377, 416, 419). This confusion was caused by the similarities between the nearly identical plasmids. Both plasmids contain the selectable marker *hpt*, as well as GUS and GFP reporters. The correct gene order of the reporters in pCAMBIA1304 is *gfp-gusA*.

Line 400-412 Fig 4 (now Figure 3) in this paragraph is Fig 1.

Response: We are not sure what the reviewer is referring to.

Line 440-441 the expected 600 bp and 235 bp.

After the correction of cleavage site, why the size of these two fragments are unchanged?

Response: We believe the reviewer has misunderstood the nature of the experiment. The goal of the experiment was to verify that the designed sgRNA when assembled *in vitro* with Cas9 to form an RNP complex is capable of successfully cleaving the target site in an 835 bp PCR-amplified fragment of the CA-B4 gene during *in vitro* incubation. The results clearly show the RNP complex can cleave the 835 bp target site fragment into 600 bp and 235 bp fragments.

Line 468-469 “a nucleotide substitution…a nucleotide substitution…”

To be more precise, I would suggest “one nucleotide deletion…one nucleotide insertion…”.

Response: We have modified the wording as requested (Lines 550-552).

Line 539-540 Cas9/sgRNA RNP-directed indels precisely at the site of Cas9/sgRNA cleavage.

The description is very likely to be true but a bit too strong to use “precisely”. Unfortunately, because the insertion or deletion are located at the original TTTT sequence, it is indistinguishable the insertion/deletion are occur at the last T next to cleavage site or are occur at other positions.

Response: We removed “precisely” from the sentence (Line 660).

Please use consistent name and style of gfp-gusA for gene or GFP-GUS reporter for protein. e.g. Line 162: gfp:gusA fusion reporter (:?); Line367: GFP-GUS reporter gene (gene); Line393: gfp-gusA reporter(no italic).

Response: We agree and now reference *gfp-gusA* consistently (Lines 161, 393, 412).

Cas9/gRNA or Cas9/sgRNA are both fine. But I would suggest to use consistent name in an article.

Response: We agree and now reference Cas9/sgRNA consistently (Lines 129, 115, 233, 235, 252, 527, 533, 563, 600).

Similar issue, CRISPR/Cas9 in reference list are all showing as Crispr/Cas9? I checked some PLOS ONE articles and their reference list still used CRISPR/Cas9 or CRISPR/CAS9. I don’t think using CRISPR violate the style of PLOS ONE reference format

Response: We replaced all Crispr/Cas9 references with CRISPR/Cas9 (Lines 899, 903, 907, 913, 971).

Fig 5 (now Figure 4). The sequences of virus and ODN could be aligned better.

Fig 5 (now Figure 4). The marker for sgRNA, gray color is correct but line art is one-nucleotide longer.

Response: Both corrections were made to Figure 4.

**Reviewer #2**:

The manuscript was modified with some improvements. However, there are still a lot of major problems, including those I missed in the last time. The manuscripts must be largely improved to fulfill the minimum requirements for scientific publication.

***Major points:***

Line 381–383: GFP expression is not fully supported by the provided data, considering the following statement. It is strange only GFP was expressed at protein level and GUS was not. The result implies that GFP observed in Figure 3 is autofluorescence or artificial. To validate GFP expression, authors must provide the image of negative control.

Response: We have removed the original Figure 3 from the manuscript.

Line 410: In the manuscripts, inhibition effect is described with two levels, “the greatest negative effect” or “minor growth inhibition”. This description is not consistent with three-level description in the table legend, “complete”, “moderate”, and “slight”. They must be consistent. Also, if the effect is judging from the size of spots, actual size must be provided as numerical number. At least, photo image must be provided as a supplemental figure.

Response: As explained in the text and in our previous response to the reviewer’s comments, the goal of the experiment was to gain a rough estimate of growth inhibition caused by various potential cell wall degrading enzymes. In most cases (as shown in Table 1) the results were starkly different between treatments. The results provided clear guidance regarding which treatments were best to use. The report, as it stands, provides the reader with information needed if they wish to use a different cell wall degrading enzyme than the one which we chose.

Line 476–482: Total number of plaques sequenced in each attempt must be precisely presented as Table. Also, the number of mutations found in those sequences must be provided.

Response: We have added the size of the 835 bp 034r gene fragment to line 557 of the text (i.e., “----- involved in DNA sequencing of the 835 bp region of interest.”) With this new information, the statement on lines 556-560 provides the reader with a complete view of the situation. That is, we found mutations only at or near the Cas9/sgRNA cut site and nowhere else in the 835 bp fragment from over 50 plaques.

Line 478–482: Basically, it is impossible to discriminate NHEJ-induced mutations from natural mutation. Therefore, it is important to show the mutation rate in Cas9-cleavage site is higher than other sites, and authors’ description, ‘no supposedly “spontaneous” mutations were discovered at or near the target site for Cas9/sgRNA cleavage located 3 nucleotides upstream of the PAM site’ is not response to the suspicion.

Response: All references to a prospective NHEJ event have now been indicated as only possibilities (Line 673), not conclusions. The comments in the response immediately above (as well as the information provided in the text) make it abundantly clear to the reader that the only mutations we observed were at or within 3 bp of the Cas9/sgRNA cut site. The lack of mutations elsewhere in the thousands of base pairs of sequenced DNA make the likelihood of the observed mutations being spontaneous exceedingly low.

The authors must provide the number of mutations found in target and non-target sites in all the attempts. Also, all the sequence must be provided at least for the first experiment as a supplemental figure.

Response: Please refer to the previous two responses.

Line 579–580: Judging from the manuscripts, only a part of 034r gene was sequenced. How do they conclude that the wildtype 034r gene was intact?

Response: We clarified we can only confirm domain 1 of *034r* is intact (Lines 703 and 711).

Line 590–591: If the authors think the Agrobacterium-mediated method is promising, why did they give up the method and move to enzymatic method?

Response: Please refer to our response to the first comment of Reviewer #1.

***Minor points:***

Overall, they should use more scientific terms and phrases, and provide “necessary and sufficient” information with clear separation into Introduction, Materials and Method, Results, and Discussion.

Line 17: The sentence should start from “Genetic and molecular modifications…”

Response: “The ability to carry out” was removed (Line 17).

Line 26: Does T-DNA mean the T-DNA region in Ti plasmid?

Response: Yes, the transfer DNA (T-DNA) is the DNA transferred from the tumor-inducing (Ti) plasmid from *A. tumefaciens* and inserted into the host organism. We have inserted the word “*Agrobacterium”* before “T-DNA” to clarify this for the reader (Line 25).

Line 27: “and gene silencing…of this system” should be removed.

Response: “and gene silencing…of this system” was removed (Line 26).

Line 27: The sentence should start from “To develop…”

Response: “In effort” was removed (Line 26).

Line 68–99: These three paragraphs have redundant and irrelevant information, so they should be reorganized.

Response: We believe the three paragraphs contain exactly the kind of information that an introduction is expected to provide to readers in a form that is relatively complete and easy to follow.

Line 94–99: This information should be moved to results or methods.

Response: We believe this information provides the reader a general summary of our work without providing any specific results or methods (Lines 99-104).

Line 123: “(caused by Cas9/gRNA-directed gene editing)” should be removed, because this information is misleading. The authors’ method cannot discriminate Cas9-induced mutation from natural mutation.

Response: We have changed the wording to “likely caused by Cas9/sgRNA-directed gene editing.” (Line 128). As detailed in our two earlier responses to this issue (see “Lines 556-560” above), the chances that the observed mutations being due to random mutations are exceedingly small and, for this reason, we believe the revised statement is reasonably conservative.

Line 108–124: This information should be moved to results or methods.

Response: After careful review of this matter, we think the present location of this information is appropriate.

Line 125–146: Information in this paragraph should be more generalized to explain the rational of CRIPSR-Cas9 system.

Response: We believe added information would be unnecessarily repetitive. We describe the general use of CRISPR in previous alga species in prior paragraphs and also elaborate more on the topic in the discussion section.

Line 149–152: In description of chemical concentration, hydrated water should be removed.

Response: hydrated waters were removed (Lines 154-157).

Line 164: What is CaMV?

Response: The 35S promoter from the plant pathogen Cauliflower Mosaic Virus (CaMV) is a commonly used promoter in driving constitutive expression of transgenes. It has now been defined as such in the text.

Line 181: “accessed” should be changed to “counted”.

Response: “accessed” was changed to “counted” (Line 199).

Line 199–200: Information on the place of microscope should be separated from that on manufactures.

Response: This information has been removed (Line 217).

Line 244: Show exact sequence of the primers.

Response: Custom primer sequences were previously supplied in the legend of Fig. S1. However, we relocated the primer sequences inside the text (Lines 271-273) and removed them from Fig. S1.

Line 240: What is GENEWIZ? Is it company or machine for sanger sequence?

Response: GENEWIZ is a company that offers sanger sequencing services. For clarification, we added the company’s address in the text (Line 274).

Line 249: Agar concentration should be shown as percentage.

Response: Agar concentration was changed to percent (Line 278).

Line 257: What is the purpose of a solution?

Response: NaCl is one component of NC64A’s growth media (MBBM), and CaCl_2_ promotes uptake of materials into cells.

Line 259: A word, “previously” should be removed.

Response: “previously” was removed (Line 288).

Line 269: A phrase “by NHEJ” should be removed. PCR and sequence cannot be discriminate mutations by NHEJ from those by other events.

Response: “by NHEJ” was removed (Line 297).

Line 270: A word “first” should be removed.

Response: “first” was removed (Line 298).

Line 291: A phrase “by NHEJ” should be removed. PCR and sequence cannot be discriminate mutations by NHEJ from those by other events.

Response: We have substituted the phrase “by NHEJ and/or other error-prone DNA repair mechanisms” (Line 324).

Line 301: A phrase “by NHEJ” should be removed. PCR and sequence cannot be discriminate mutations by NHEJ from those by other events.

Response: “by NHEJ” was removed (Line 334).

Line 314–316: This is a result.

Response: We moved these remarks to the results section (Lines 487-495).

Line 323: A phrase “by NHEJ” should be removed. PCR and sequence cannot be discriminate mutations by NHEJ from those by other events.

Response: “by NHEJ” was removed (Line 359).

Line 327: “tumefaciens” should be italic.

Response: “tumefaciens” has been italicized” (Lines 363 and 365).

Line 332–333: The growth seemed to be inhibited at 100 mg/L in Figure 1A.

Response: We revised this sentence to “Growth of *A. tumefaciens* was inhibited at a cefotaxime concentration of 100 mg/L (Fig 1A) whereas the growth of NC64A was found to be uninhibited in cefotaxime-supplemented media at the same concentration and up to at least 1000 mg/L (Fig 1B).” (Lines 368-371).

Line 343–344: The Y-axis label of B and C should be the same.

Response: The Y-axis label of panels B and C (Figure 1) are now the same.

Line 347–351: Schematic image of the plasmid construction should be provided.

Response: We believe a schematic image of the vector is not needed. The structure of this commonly used vector has been published and is readily available online.

Line 370: Why are the sequences of hpt and gfp-gusA different from original sequence? Sequence of the original plasmid constructed in this study must be checked.

Response: The original plasmid sequence has been verified.

Line 378–379: Figure 2C is not required.

Response: Figure 2C was removed.

Line 382: Fluorescent microscopy confirms expression of GFP protein.

Response: This section has been removed from the manuscript.

Line 398: The bar should be precisely 5 µm.

Response: The figure was removed.

Line 405: A word “greatest” should be modified.

Response: The word, “greatest”, was replaced with “complete growth inhibition” which in now consistent with Table 1 (Line 428).

Line 420–423: These percentage should be presented in Figure 4 (now Figure 3).

Response: Percentages are now included in the Figure 3 legend (Lines 499-501).

Line 439–441: The intensity of cleaved band is too faint. Authors must present clearer image for this experiment.

Response: Although faint, the band in question is undoubtedly present. The question being asked is “is the fragment cut?” The answer provided in the figure is clearly “yes”.

Line 467: Show exact sequence of the primers.

Response: Primers are now provided (see above).

Line 472–476: This part should be moved to discussion.

Response: This part was moved to the discussion (Lines 659-664).

Line 482–484: This part should be moved to discussion.

Response: This part was moved to the discussion (Lines 664-666).

Line 498–499: “site-directed mutagenesis by preassembled Cas9 protein-sgRNA RNPs” cannot be verified only by the sanger sequence results.

Response: We added the pretense, “to support the possibility of site-directed mutagenesis…” (Line 597).

Line 505: A word “also” should be removed.

Response: “also” was removed (Line 603).

Line 508: A phrase should be “MscI site is intact”

Response: “MscI site is intact” was added (Line 606).
